# Müller glia as an important source of cytokines and inflammatory factors present in the gliotic retina during proliferative vitreoretinopathy

**DOI:** 10.1002/glia.22942

**Published:** 2015-11-10

**Authors:** K. Eastlake, P. J. Banerjee, A. Angbohang, D. G. Charteris, P. T. Khaw, G. A. Limb

**Affiliations:** ^1^ Department of Ocular Biology and Therapeutics, UCL Institute of OphthalmologyLondonUnited Kingdom; ^2^ NIHR Biomedical Research Centre at Moorfields Eye Hospital and UCL Institute of OphthalmologyLondonUnited Kingdom

**Keywords:** Müller glia, PVR, retinal gliosis, cytokines, retina degeneration

## Abstract

Retinal gliosis is characterized by biochemical and physiological changes that often lead to Müller glia proliferation and hypertrophy and is a feature of many neuro‐degenerative and inflammatory diseases such as proliferative vitreoretinopathy (PVR). Although Müller glia are known to release inflammatory factors and cytokines, it is not clear whether cytokine production by these cells mirrors the pattern of factors present in the gliotic retina. Lysates from normal cadaveric retina and gliotic retinal specimens from patients undergoing retinectomy for treatment of PVR, the Müller cell line MIO‐M1 and four human Müller glial cell preparations isolated from normal retina were examined for their expression of cytokines and inflammatory factors using semi‐quantitative dot blot antibody arrays and quantitative arrays. Comparative analysis of the expression of inflammatory factors showed that in comparison with normal retina, gliotic retina exhibited greater than twofold increase in 24/102 factors examined by semiquantitative arrays, and a significant increase in 19 out of 27 factors assessed by quantitative methods (P < 0.05 to *P* < 0.001). It was observed that with the exception of some chemotactic factors, the majority of cytokines and inflammatory factors were produced by Müller glia *in vitro* and included G‐CSF, MCP‐1, PDGF‐bb, RANTES, VEGF, and TGFβ2. These results showed that a large number of inflammatory factors expressed by Müller glia *in vitro* are upregulated in the gliotic retina, suggesting that targeting the production of inflammatory factors by Müller glia may constitute a valid approach to prevent neural damage during retinal gliosis and this merits further investigations. GLIA 2016;64:495–506

## Introduction

Events leading to the development of PVR, a common complication of retinal detachment, have been associated to those of the inflammatory and wound healing responses (Garweg et al., 2013; Oberstein et al., 2011). Cellular processes leading to the development of PVR involve migration and proliferation of a variety of cells including retinal pigment epithelium (RPE) (Hiscott et al., 1999), mononuclear leucocytes (Hiscott et al., 1989; Limb et al., 1993), microglia (Weller et al., 1991a, 1991b), and Müller glia (Bringmann et al., 2009), all of which are known to contribute to inflammation by releasing proinflammatory factors and cytokines. Although retinal pigment epithelial (RPE) cell proliferation was thought for a long time to be the major player in the development of PVR (Kirchhof and Sorgente, 1989; Palma‐Nicolas et al., 2010; Parrales et al., 2013), in recent years it has been accepted that Müller glia also play a very important role in the pathogenesis of this condition (Bringmann et al., 2009; Charteris et al., 2007; Guidry, 2005; Lewis et al., 2010; Morescalchi et al., 2013; Velez et al., 2012).

Müller glia span across the whole width of the retina and provide structural and metabolic support to retinal neurons (Bringmann and Wiedemann, 2012). Whilst there is evidence that Müller glia become progenitor cells in fish and amphibians in response to retinal damage (Lenkowski et al., 2013), in the adult mammalian retina this feature appears to have been lost (Loffler et al., 2015). In contrast, reactive Müller cell gliosis characterized by morphological, biochemical, and physiological changes, often occurs in the mammalian retina in response to injury (Bringmann et al., 2006). This process is thought to develop as a protective mechanism to prevent further damage to the retina and to promote tissue repair. Yet, it does not appear to be beneficial in the adult mammalian retina and it has been thought that the release of proinflammatory cytokines and growth factors from Müller glia, can lead to further degeneration (Bringmann and Wiedemann, 2012).

Several growth factors, cytokines, and matrix degrading enzymes are observed in vitreous, subretinal fluid, and retinal tissue from eyes affected by PVR (Lei et al., 2010; Limb et al., 1994, 1991; Symeonidis et al., 2014). Infiltrating macrophages and local microglia are thought to secrete growth factors, which in turn promote further cytokine production and cellular migration and differentiation (Weller et al., 1990), whilst RPE cells have been thought to be responsible for the production of extracellular matrix (Hiscott et al., 1999), as well as various proinflammatory cytokines, including transforming growth factor β2 (Hirsch et al., 2015). Müller glia have been shown to release several inflammatory factors and cytokines (Bringmann et al., 2009) and some cytokines in turn have been shown to stimulate production of other cytokines by Müller glia (Yoshida et al., 2001). In addition, Müller glia express toll‐like receptors (TLRs) (Kumar et al., 2013) and receptors for advanced glycation end products (RAGE) (Zong et al., 2010) that upon binding to their ligands induce production of proinflammatory cytokines, chemokines and neuroprotective growth factors by these cells.

Although various cytokines and growth factors have been identified in vitreous and retinal tissues from many retinal conditions associated with gliosis (Chua et al., 2012; Franks et al., 1992; Limb et al., 1994, 1991; Muether et al., 2013; Suzuki et al., 2011), it is not clear to what extent Müller glia may contribute to the release of factors present in the gliotic retina, and whether the pattern of cytokine expression in the gliotic retina may mimic that of isolated Müller cells. It was therefore the aim of this study to investigate the expression of a range of proinflammatory factors in Müller glia *in vitro* and to examine whether this expression parallels that seen in the gliotic retina from patients with proliferative vitreoretinopathy (PVR).

## Materials and Methods

### Tissue and Cell Culture

Four retinal specimens isolated from normal cadaveric donors were obtained from Moorfields eye Bank, with prior consent for research. All eyes were obtained within 24‐h post mortem and the age range of the donors was 34–88 years. The eyes were kept in sterile saline and retinas carefully removed and washed in PBS. Specimens for protein analysis were obtained by excising sections of peripheral retina between 1–3 mm × 1–5 mm (3–5 mm^2^) to match the size of the retinectomy specimens obtained. Samples were then frozen at −80°C until use. Six peripheral retinectomy specimens (3–5 mm^2^) from eyes undergoing retinal surgery for treatment of proliferative vitreo‐retinopathy (PVR) were obtained from Moorfields eye Hospital, upon written consent from the patients. The age of the patients ranged between 58 and 71 years, with a duration of PVR of 2–10 weeks. All tissues used in this study were obtained and treated according to guidelines from the Local Ethics Committee at Moorfields and the Institute of Ophthalmology and followed the tenets of the Declaration of Helsinki. Isolated retinas were washed in PBS and frozen at −80°C until use.

The Müller cell line (MIO‐M1) established in our laboratory and derived from normal retinae (Limb et al., 2002), and other four Müller cell preparations isolated as previously described (Limb et al., 2002) were used in the study. These were named 6387, 6391, 6390, and 6426. Although they were used between passages 9 and 14, these cells have the characteristics of the MIO‐M1 cell line, and upon further passages they have been shown to be spontaneously immortalized. Each cell preparation was grown to a confluent monolayer on plastic flasks in DMEM containing 10% FCS. Monolayers were washed in PBS and detached from tissue culture flasks using a cell scraper. Cells were resuspended in PBS and centrifuged to obtain a cell pellet. This was then frozen at −80°C until use.

### Preparation of Retina and Cell Lysates

Cell lysis was carried out in retinal specimens and Müller cell pellets using a BioPlex cell lysis kit (171‐304011, BioRad, UK) according to the manufacturer's instructions. Briefly, samples were rinsed with cell wash buffer, and homogenized in 500 μL cell lysis solution (containing 500 mM PMSF). Samples were then frozen at −70°C, thawed and sonicated on ice followed by centrifugation at 4,500*g* for 4min (cell lysates) or 20 min (retina samples). Supernatants containing proteins were collected and protein concentrations were determined using a BCA assay kit (Thermo Fischer, UK).

### Proteome Profiler Antibody Array

The R&D Systems Human XL cytokine array kit (ARY022, R&D Systems, UK) was used to perform a general semi quantitative analysis of various cytokines expressed in normal and gliotic human retinal lysates as well as cultured Müller glia lysates. Protocols were followed as per manufacturer's instructions. Because of the small size of the gliotic and normal retinal specimens investigated (3–5 mm^2^), it was necessary to pool the protein lysates of gliotic or normal retina to yield the protein concentrations of 150 μg mL^−1^ required for the assay. A pool of Müller cell lysates was also made in order to undertake a comparative analysis between samples.

Protein extracts of cell and retinal samples were incubated with the antibody array membranes overnight at 4°C. After washing, membranes were incubated with detection antibodies and chemiluminescent reagents provided in the kit. Membranes were protected in plastic sheeting before imaging using an autoradiography cassette and X‐ray film. Spot intensity analysis was carried out using ImageJ and Microsoft Excel.

### Quantitative Analysis of Cytokines and Growth Factors

The BioPlex‐pro 27 plex immunoassay (BioRad, UK), which provides quantitative values, was used in this study to confirm results from the proteome profiler array. Experiments were carried out following the manufacturer's instructions. Using the protein standards provided, six gliotic retinal samples, four normal cadaveric retinae, and five different Müller cell preparations including the MIO‐M1 cell line, were each prepared to contain between 200 and 900 μg mL^−1^ protein and individually examined in the immunoassay. In addition, each individual sample was assessed in duplicate. The magnetic bead stock was diluted 1:20 with assay buffer and 50 μL of this solution was loaded into each well of a 96‐well plate. Beads were washed twice in wash buffer using a Bio‐Plex® Handheld Magnetic Washer (#171‐020100, BioRad). The standards and samples were then loaded in duplicate into the wells and the plate was sealed and incubated at room temperature for 2 h. After the incubation, the plate was rinsed with wash buffer three times using the magnetic plate, before addition of 25 μL of detection antibodies. After 1‐h incubation on the shaker, the plate was washed three times, and 50 μL of streptavidin added to each well. The plate was then incubated for 30 min on the shaker and washed three times. About 125 μL of assay buffer was added to each well and the plate incubated for 30 s before reading on the BioPlex Magpix™(BioRad,UK) system. Cytokines detected in this array included FGFb, Eotaxin, G‐CSF, GM‐CSF, IFN‐γ, IL‐1β, IL‐1ra, IL‐2, IL‐4, IL‐5, IL‐6, IL‐7, IL‐8, IL‐9, IL‐10, IL‐12 (p70), IL‐13, IL‐15, IL‐17, IP‐10, MCP‐1, MIP‐1α, MIP‐1β, PDGF‐BB, RANTES, TNF‐α, and VEGF. Tests were also performed to analyse the three isoforms of TGF beta, using the BioPlex‐pro TGFbeta 3‐plex immunoassay (BioRad,UK). Because TGFβ is normally produced as a high molecular weight protein complex consisting of a mature TGFβ dimer, the latency‐associated peptide (LAP) and the latent TGFβ binding protein (LTBP), transient acidification followed by neutralization is needed to release the immunoreactive form for quantification. Because this treatment affects the quantification of most cytokines, it is necessary to undertake specific assays for TGFβ (Kropf et al., 1997). Therefore, for the TGFβ analysis, lysates of six gliotic retina, four normal retina, or Müller cells were activated with 1N HCl, followed by neutralization with 1.2N NaOH/0.5 M HEPES buffer prior to performing the assay described above. The same procedure was followed to assess the cell culture supernatants of MIO‐M1 cells, which were examined as single specimens. Mean cytokine values of individual samples were obtained using the BioPlex manager 6.1 (BioRad,UK) and Microsoft Excel programmes. Statistical comparison between different groups was determined by two tailed, unpaired *t* tests, using the GraphPad Prism5 programme.

## Results

### Expression of Cytokines and Growth Factors by Müller Glial Cell Preparations

**Figure 1:**
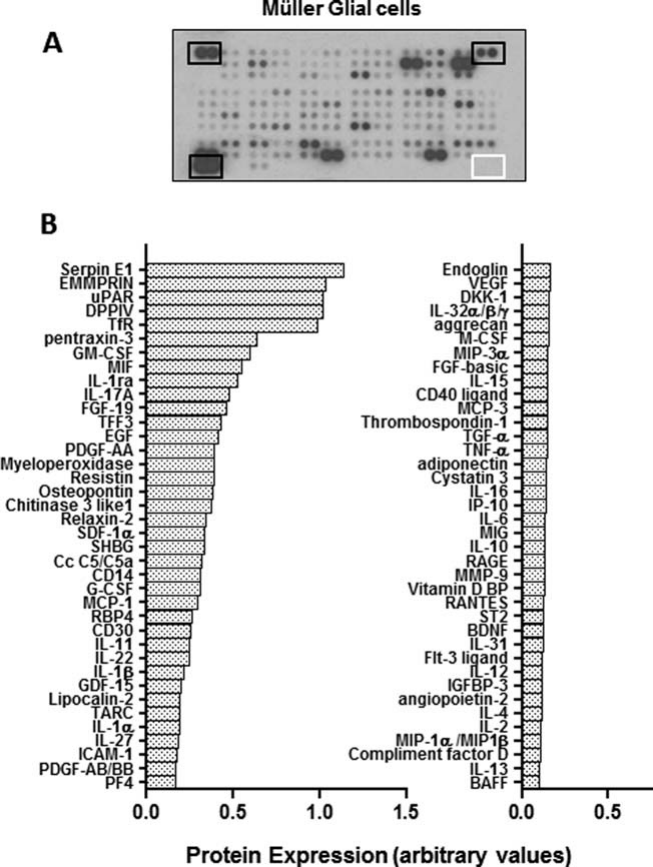
Expression of cytokines and growth factors by Müller glia *in vitro*. Images show the relative expression of cytokines and growth factors detected in a pooled lysate from the five different Müller glia cell lines investigated. (**A**) Dot blot membrane array shows the intensity of expression of the various factors in the cell lysate. Spots marked by black boxes indicate positive controls, whilst spots marked by the white box indicate the negative controls. (**B**) Histograms show in descending order the relative levels of cytokines and growth factors expressed by Müller glia. Values were normalized to the positive controls. Relative values below 0.1 were excluded from the analysis.

Semiquantitative analysis of a pool of lysates from the five different Müller cell preparations used in the study detected the presence of 76 factors out of the 102 examined. The cytokines and growth factors predominantly observed were those associated with retinal differentiation (EGF), axon regeneration (osteopontin) proliferation (DPPIV, MIF, EGF, PDGF‐aa, FGF‐19) matrix organization (Serpin E1, EMMPRIN, uPAR, DPPIV), inflammation (Pentraxin‐3, GM‐CSF, MIF, IL‐1RA, IL‐17a), and apoptosis (GM‐CSF, myeloperoxidase, MIF, SerpinE1). Basal levels of other proinflammatory cytokines and chemokines including IL‐6, M‐CSF, MIP‐3a, IL‐15, MCP‐3, TNFα, MIP‐1a/b, and matrix components such as thrombospondin were also detected at very low levels (Fig. 1).

**Figure 2:**
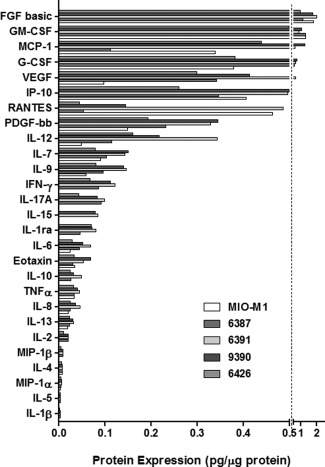
Expression of cytokines and growth factors by Müller glia *in vitro* as determined by quantitative analysis. Histogram shows the quantitative analysis of the expression of cytokines in the five different Müller cell lysates examined. Levels are shown in descending order according to the levels observed in the majority of cells.

Further examination of the expression of cytokines in the MIO‐M1 cell line and four different Müller glial cell lysates by quantitative methods (BioPlex Pro) showed that these cells expressed all the 27 cytokines analyzed, although some cytokines were found to be present in the cell lysates at very low levels. FGFb consistently exhibited the highest expression levels in all Müller glial samples at 1–2 pg μg^−1^ protein (Fig. 2). Expression levels largely related to the semiquantitative analysis (Fig. 1) and showed high concentrations of GM‐CSF, MCP‐1, and G‐CSF. Many factors showing low expression (below 0.4 pg μg^−1^ protein) in the quantitative array also showed low expression in the semiquantitative array and included PDGF‐bb, IL‐12, IL‐15, IL‐6, IL‐10, IL‐13, IL‐2, IL‐4, IL‐17a, IL‐1b, IL‐1ra, TNF‐α, MIP‐1b, and MIP‐1a. Unlike most cytokines which were present in all the cell lines examined, IL‐15 was only observed in two out of five cell preparations.

**Figure 3:**
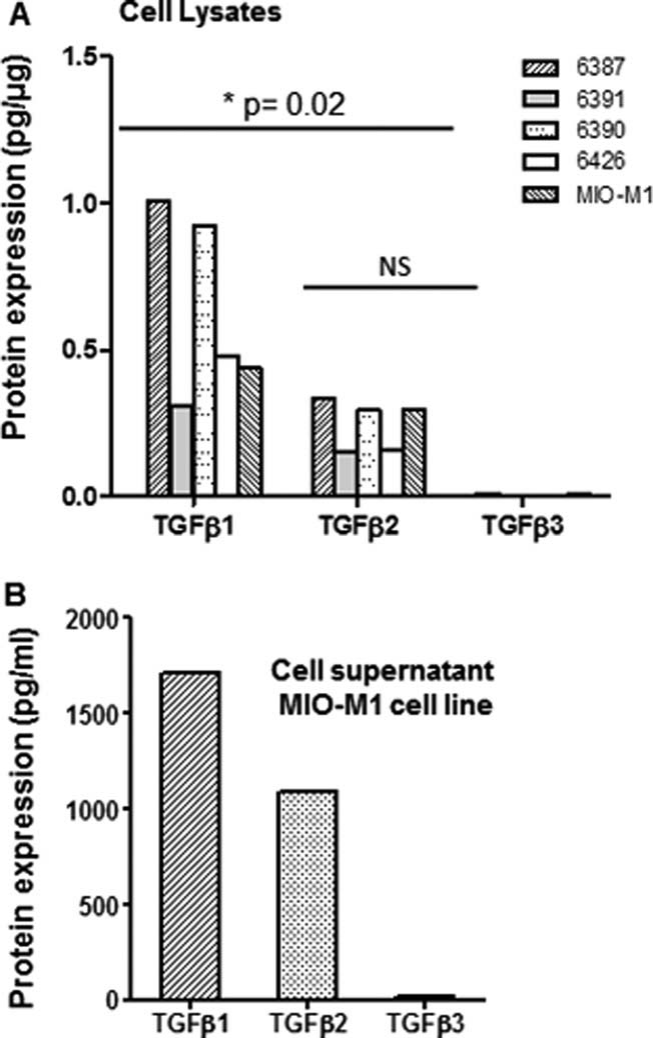
Expression of TGFβ isoforms by Müller glia *in vitro*. (**A**) Histograms show the mean value of the levels of expression of TGFβ1, TGFβ2, and TGFβ3 in each of the individual Müller glial cell lysates investigated, as determined by a quantitative immunoassay. All the cell lysates examined expressed higher levels of TGFβ1 as compared with TGFβ2 (unpaired two‐tailed *t* test, *P* = 0.02) and TGFβ3 (unpaired two‐tailed *t* test, *P* < 0.001). Very low levels of TGFβ3 were only detected in MIO‐M1 and 6387 cells. (**B**) Quantitative analysis of the TGFβ isoforms present in individual cell culture supernatant of the Müller cell line MIO‐M1 shows that TGFβ1 and TGFβ2 are released by these cells in similar proportion to that seen in cell lysates. Minimally detectable levels of TGFβ3 were released by these cells.

Quantitative analyses of five Müller glial cell lysates were performed to assess the expression of the three different isoforms of TGFβ. It was observed that all the five Müller glial cell preparations investigated expressed relatively higher levels of TGFβ1 (0.64 ± 0.33 pg μg^−1^ protein) than TGFβ2 (0.23 ± 0. 0.1 pg μg^−1^ protein) (*P* = 0.02) or TGFβ3 (0.01± 0.001 pg μg^−1^ protein) (*P* < 0.001) (Fig. 3A). TGFβ3 was only observed above detectable levels in two out of the five cell preparations analyzed (MIO‐M1 and 6387). Quantification of TGFβ protein isoforms 1, 2, and 3 in a culture supernatant of the MIO‐M1 cell line showed that TGFβ1 was the predominant isoform released (1713 pg mL^−1^), followed by TGFβ2 (1093 pg mL^−1^). In contrast, very low levels of TGFβ3 (15.25 pg mL^−1^) were observed in the supernatant of these cells (Fig. 3B). The levels of TGFβ isoforms observed in the supernatant of MIO‐M1 cells were related to the levels of these cytokines detected in the cell lysates (Fig. 3A).

### Comparison of the Expression of Cytokines and Growth Factors Between Gliotic Retina of Proliferative Vitreoretinopathy and Normal Retina

**Figure 4:**
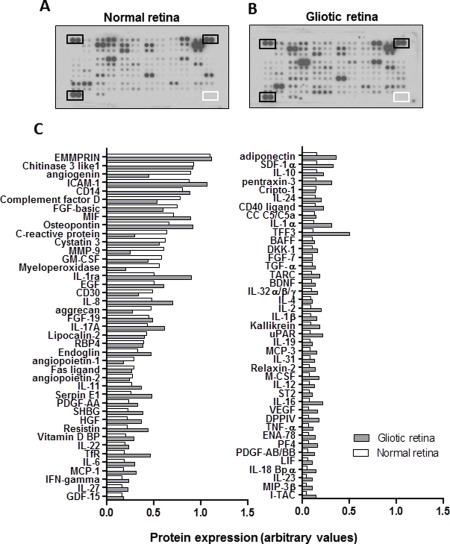
Semi‐quantitative analysis of the expression of cytokines and growth factors in gliotic and normal human retina. Images show the relative expression of cytokines and growth factors detected in pooled lysates from four normal cadaveric retina and six gliotic retinectomy specimens. (**A**, **B**) Dot blot membrane arrays show the intensity of expression of various factors in the retinal lysates. Spots marked by black boxes indicate positive controls, whilst spots marked by the white box indicate the negative controls. (**C**). Histograms show in descending order the levels of expression of the various factors in the pooled specimens. Values were normalized to the positive controls. Relative values below 0.1 were excluded from the analysis.

Semiquantitative analysis of a pool of six PVR specimens and four retinal fragments from normal cadaveric retina detected the presence of 82 factors out of 102 examined (Fig. 4). All the cytokines and growth factors identified in the normal retina were also present in the gliotic retina. However, 21 of these factors were found >2‐fold upregulated in the gliotic retina as compared with normal retina, whilst five of these factors were <0.6‐fold downregulated in the gliotic retina as compared with normal retina (Table 1). Factors including TFF3, I‐TAC, and IL‐16 exhibited the highest fold increase in the gliotic retina when compared with normal retina (Table 1). As expected, inflammatory cytokines such as IL‐1a, VEGF, PF4, IL‐16, PDGF‐AA/BB, M‐CSF, adiponectin, MIP‐3b, IL‐18bpa, I‐TAC, and IL‐2 also exhibited a two‐fold increase in the gliotic retina compared with normal retina. Factors involved in metabolic regulation such as adiponectin was also upregulated more than two‐fold in the gliotic retina as compared with normal retina. Factors found to be two‐fold downregulated in the gliotic retina compared with normal retina included angiogenin, C‐reactive protein, myeloperoxidase, MMP‐9 and aggrecan (Table 1).

**Table 1:** Semi‐quantitative Analysis of the Expression of Cytokines and Inflammatory Factors in PVR Retina as Compared with Normal Retina

Factor	Function	Fold change (gliotic/normal retina)
TFF3	Cell growth, metastasis, and angiogenesis	4.623920
I‐TAC	Chemokine	3.725999
IL‐16	Cytokine/chemoattractant	3.209796
IL‐18 Bpa	Inhibits IL‐18	2.975417
DPPIV	Antigenic enzyme	2.748755
uPAR	Glycoprotein/receptor	2.747772
IL‐1a	Pro‐inflammatory cytokine	2.611321
PF4	Chemokine–blood coagulation	2.595450
MIP‐3b	Chemokine	2.530014
TfR	Carrier protein; iron import	2.487686
VEGF	Growth factor–vasculogenesis	2.460055
IL‐23	Pro‐inflammatory cytokine	2.435597
M‐CSF	Pro‐inflammatory cytokine	2.371067
adiponectin	Hormone; glucose regulation	2.357242
IL‐2	Pro‐inflammatory cytokine	2.259597
ENA‐78	Chemokine	2.237109
PDGF‐AB/BB	Growth factor	2.211187
SDF‐1a	Chemokine	2.185202
pentraxin‐3	Immune regulation	2.175439
Kallikrein	Enzyme; proinflammatory	2.107335
Resistin	Hormone; proinflammatory	2.049007
aggrecan	Cartilage proteoglycan	0.575574
angiogenin	Enzyme; angiogenesis	0.500763
C‐reactive protein	Proinflammatory	0.466765
MMP‐9	Matrixin enzyme	0.408317
Myeloperoxidase	Lysosomal enzyme	0.366569

Values obtained from semiquantitative analysis of dot blot arrays showing the fold change in the expression of cytokines and growth factors in the gliotic retina as compared with the normal retina. Values below 0.1 were excluded from analysis.

**Figure 5:**
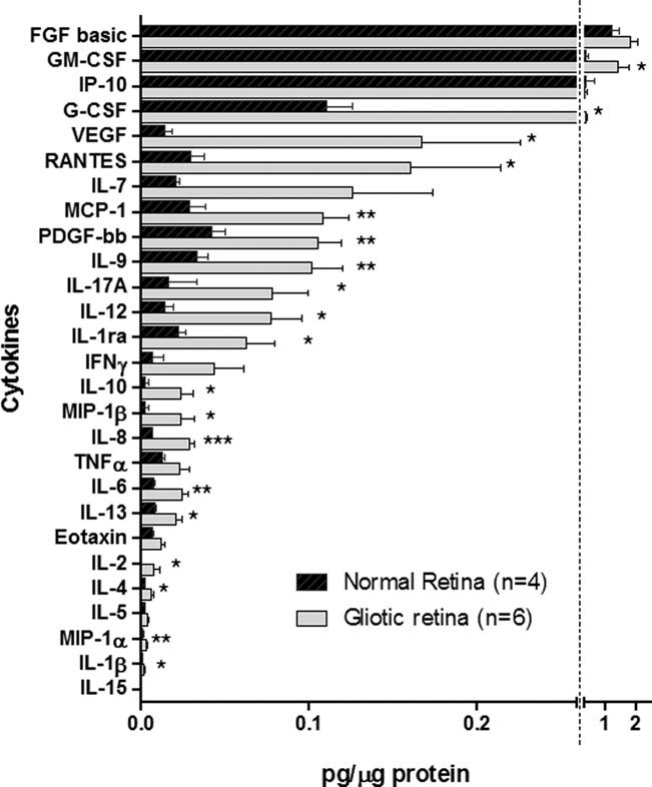
Expression of inflammatory cytokines in the gliotic and normal human retina. Histogram shows a quantitative analysis of the expression of cytokines arranged in a descending order according to the levels observed in the gliotic retina. Asterisks denote a significant upregulation in the gliotic retina (red columns) as compared with the normal retina (blue columns). Error bars indicate the mean ± SEM for each group. *P < 0.05; **P < 0.01; ***P < 0.001. *n* = number of samples investigated.

Further analysis by quantitative methods compared the levels of 27 cytokines and growth factors between lysates derived from normal cadaveric retina (*n* = 4) and gliotic retina obtained from patients with PVR (*n* = 6). FGF basic, GM‐CSF and IP‐10 showed high expression in the retinal lysates in comparison to other cytokines analyzed. No expression of IL‐15 was detected in any of the retinal lysates analyzed. A significant increase, was observed in 19 out of the 27 cytokines investigated quantitatively in the gliotic retina as compared with the normal retina and included GM‐CSF, G‐CSF, VEGF, RANTES, MCP‐1, PDGF‐bb, IL‐9, IL‐17a, IL‐12, IL‐1ra, IL‐10, MIP‐1b, IL‐8, TNFα, IL‐6, IL‐13, IL‐2, IL‐4, MIP‐1a, and IL‐1b (*P* < 0.05 to *P* < 0.001; two tailed *t* test) (Fig. 5).

**Figure 6:**
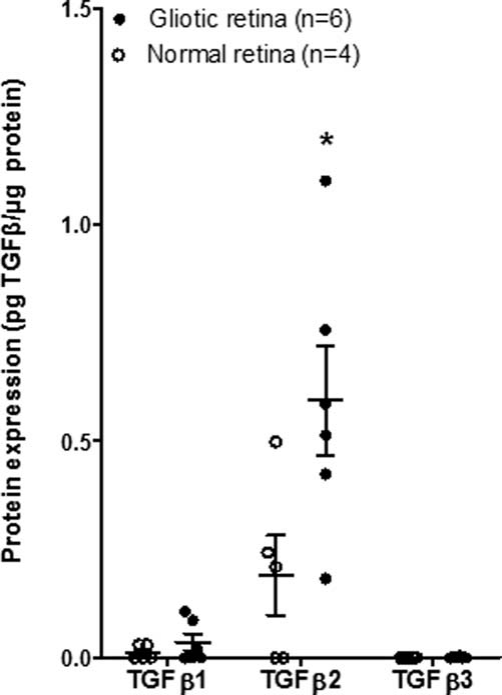
Levels of TGFβ isoforms in gliotic and normal human retina. Scatter dot‐plots show the levels of expression of the three different isoforms of TGFβ in four normal and six gliotic human retinal specimens. Error bars indicate the mean ± SEM for each group. * Unpaired two‐tailed *t* test, *P* < 0.05 vs. normal retina; ** unpaired two‐tailed *t* test, *P* < 0.01 v. normal retina.

Quantitative expression of the three different TGFβ isoforms was compared between six gliotic retinal specimens and four normal retinae. TGFβ2 was the predominant isoform present in the lysates from both gliotic and normal human retinae (Fig. 6). TGFβ2 was the only isoform to be significantly increased in the gliotic retina as compared with the normal retina (*P* < 0.01). Although an increase in the levels of TGFβ1 was observed in the gliotic retina as compared with normal retina these levels were not significantly different. In addition, TGFβ3 which was not observed in the normal retina, was observed in two of the six gliotic specimens analyzed (Fig. 6).

### Comparison of the Expression of Cytokines Between Müller Glia and Gliotic or Normal Retina

**Figure 7:**
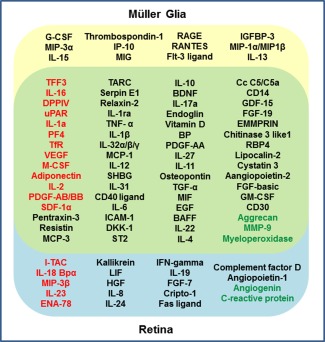
Comparison of cytokine expression between Müller glia and gliotic or normal retina. Venn diagram shows the overlapped expression of cytokines between Müller glia, gliotic, and normal retina as detected by semi‐quantitated analysis. Factors listed in the yellow section indicate those expressed by Müller glia; factors listed in the green (overlapping) section indicate those identified in Müller glia and normal and gliotic retina, whilst factors listed in the blue section indicate those only detected in normal and gliotic retina. Factors highlighted in red indicate those which were >2‐fold upregulated in the gliotic retina as compared with normal retina, whilst factors highlighted in green represent those found <0.5‐fold downregulated in gliotic retina as compared with normal retina.

Semiquantitative analysis revealed various similarities and differences in the expression of cytokines and growth factors between Müller glia and retinal specimens. Of the 95 proteins identified by the protein array, 76 (80%) were detected in Müller glia *in vitro*, whilst 83 (87%) were detected in the normal and gliotic retina. Out of the 76 proteins expressed by Müller glia, only 12 (16%) were unique to these cells, whilst 64 (84%) were also found in retinal specimens. Of the 83 proteins detected in retina, only 19 (22.9%) were unique to gliotic or normal retina (Fig. 7). Factors including TFF3, IL‐16, DPPIV, uPAR, IL‐1a, PF4, TfR, VEGF, M‐CSF, adiponectin, IL‐2, PDGF‐AB/BB, and SDF‐1a, were observed two‐fold or more upregulated in the gliotic retina as compared with normal retina, and shared their expression with Müller glia (Fig. 7). Other proteins found to be highly expressed in the Müller glia *in vitro*, including EMMRIN, Chitinase 3 like 1, FGFb, MIF, resistin and osteopontin were also found at high levels in both gliotic and normal retina, although there were no differences in their expression between these specimens (Figs. 1, 4 and 7). Aggrecan, MMP‐9 and myeloperoxidase which also shared expression between retina and Müller glia, were downregulated in the gliotic retina as compared to normal retina. Of the 19 factors detected only in retinal specimens, I‐TAC, IL‐18Bpa, MIP‐3b, IL‐23, and ENA‐78 were observed to be upregulated in the gliotic retina, whilst angiogenin and c‐reactive protein were observed downregulated in the gliotic retina (Fig. 7).

Quantitative analysis showed that out of 27 factors analyzed, all were detected in Müller glia *in vitro*. In addition, with the exception of IL‐15, which was only found in two Müller cell preparations, all factors were present in the retinal specimens. Of the 26 cytokines identified in retina, 19 (70%) were significantly upregulated (*P* < 0.05 to *P* < 0.001) in the gliotic retina as compared with normal retina (Fig. 5, Table 2). Interestingly, FGFb and GM‐CSF, which were highly expressed in the gliotic retina, were expressed at the highest levels in Müller cell lysates.

**Table 2:** Comparison of Inflammatory Factors Expressed by Müller Glia, Normal, and Gliotic Retina

	Average expression (pg/μg protein)		
Protein	Müller glia	Normal retina	Gliotic retina
IL‐15	0.040668	0	0
IL‐1b	0.001851	0.000607	0.001695*
MIP‐1a	0.003732	0.001224	0.003418**
IL‐5	0.002317	0.002111	0.003855
IL‐4	0.00598	0.002	0.005974*
IL‐2	0.017284	0	0.00788*
Eotaxin	0.040389	0.006458	0.01172
IL‐13	0.022528	0.007916	0.020591*
IL‐6	0.03749	0.007552	0.024154**
TNFα	0.035241	0.012741	0.022777
IL‐8	0.026798	0.006391	0.029021***
MIP‐1b	0.007141	0.002183	0.023561*
IL‐10	0.031976	0.002435	0.023479*
IFNγ	0.096418	0.006599	0.04368
IL‐1ra	0.065974	0.022479	0.062883*
IL‐12	0.139451	0.014386	0.077607*
IL‐17A	0.078565	0.016709	0.077919*
IL‐9	0.090557	0.033561	0.101902**
PDGF‐bb	0.220385	0.041872	0.105347**
MCP‐1	0.486812	0.028808	0.108224**
IL‐7	0.105637	0.020808	0.125765
RANTES	0.30209	0.029894	0.160456*
VEGF	0.281052	0.013774	0.167558*
G‐CSF	0.488854	0.11078	0.335609*
IP‐10	0.402409	0.364124	0.316521
GM‐CSF	1.056891	0.332087	1.418945*
bFGF	1.590391	1.200721	1.809477

Table shows the mean expression values of various factors in lysates of Müller glia (*n* = 5), normal retina (*n* = 4), and gliotic retina (*n* = 6). Asterisks represent the levels of significance between normal and gliotic retina (*P < 0.05; **P < 0.01; ***P < 0.001).

## Discussion

Inflammation has been implicated in the pathogenesis of retinal gliosis from various aetiologies, including PVR, diabetic retinopathy and prevalent diseases that lead to blindness, such as AMD and glaucoma (Hollborn et al., 2008; Muether et al., 2013; Suzuki et al., 2011; Tezel and Wax, 2004). Inflammatory factors and cytokines are found in the vitreous and retinal specimens of eyes affected by various retinal degenerative conditions (Chua et al., 2012; Limb et al., 1994, 1991), whilst Müller glia *in vitro* have been shown to produce various inflammatory factors associated with gliosis (Bringmann et al., 2009). Different cell types have been implicated in the development of gliosis but it is not clear whether cytokines present in the gliotic retina predominantly derive from a specific cell population. Furthermore, the pattern of expression of inflammatory factors in both, the gliotic retina of PVR and isolated Müller glia alone have not been previously examined. This study aimed to assess whether factors produced by Müller glia *in vitro* could be associated to the high levels of cytokines and inflammatory factors present in retinal specimens of patients with PVR.

Because of the size of the gliotic specimens obtained (3–5 mm^2^), it was necessary to pool the protein lysates of gliotic and normal retina to yield the required protein concentrations to undertake the protein profile array. Since we pooled the tissue samples, a pool of protein cell lysates was also made in order to undertake a comparative analysis between samples.

As identified by semiquantitative analysis the protein found to be the most highly upregulated in the gliotic PVR retina, was trefoil factor 3 (TFF3), showing a 4.2‐fold increase in comparison with the normal retina. This factor was also found to be one of the predominant factors observed in cell lysates of Müller glia. TFF3 peptides are located to mucous epithelia as well as nervous tissue and are involved in apoptosis, cell migration and immune responses (Belovari et al., 2015). In disease, TFF3 has been associated with cell growth, metastasis and angiogenesis in cancer (Babyatsky et al., 2009; Kjellev, 2009) as well as with neurodegenerative disorders such as Alzheimer's disease (Bernstein et al., 2015). This protein has never been associated to retinal gliosis and its precise role in inflammatory processes affecting the retina is not known. It is possible that it may contribute to the abnormal proliferation and immune modulation of Müller glia within the affected retina and merits further investigations. The second most upregulated factor found ion the gliotic retina was the interferon‐inducible T Cell Alpha Chemoattractant (I‐TAC), also known as Chemokine (C‐X‐C motif) ligand 11 (CXCL11) (Rani et al., 1996) showing a 3.7‐fold increase as compared with the normal retina. Because T lymphocyte infiltration is often observed in retinal membranes of PVR (Limb et al., 1993), this factor may therefore be responsible for this effect. Interestingly, this factor was not expressed by Müller glia *in vitro*, suggesting that resident or inflammatory microglia or macrophages are responsible for its production within the inflamed retina. Other cytokines found >2‐fold upregulated in the gliotic retina included those that function as chemoattractants, such as IL‐16, platelet factor 4 (PF4) and MIP‐3b, as well as proinflammatory cytokines such as IL‐1a, and VEGF. However, most chemoattractants were expressed by Müller glia at very low levels, suggesting that although Müller glia contributes to the production of these factors in PVR, other cells in the retina, including microglia may also constitute a source of these cytokines. Interestingly, the antigenic enzyme DPPIV, a membrane anchored ecto‐protease identified as the leukocyte antigen CD26 (Augustyns et al., 1999), uPAR, a glycoprotein bound to the cell membrane and a component of the plasminogen activation system (Kjaergaard et al., 2008), and the transferrin receptor (TfR), which were all present at high levels in Müller cell lysates were also found to be >2‐fold upregulated in the PVR retina.

Quantitative analyses identified high expression of FGFb, GM‐CSF, IP‐10, and G‐CSF in the normal retina, with GM‐CSF and G‐CSF showing significant upregulation in the PVR specimens. Other cytokines found in the normal retina at relatively low concentrations, such as VEGF, RANTES, IL‐7, MCP‐1, and PDGF‐bb were also significantly increased in the gliotic retina as compared with the normal retina. This is supported by several findings in the literature, which show high upregulation of these factors in PVR, and diabetic retinopathy (Mitamura et al., 2001; Suzuki et al., 2011). Although these factors were highly expressed by the five different Müller cell preparations investigated, it is known that factors such as VEGF can be produced by microglia and macrophages (Krause et al., 2014; Liu et al., 2015), whilst the chemokines RANTES and MCP‐1 can be produced by astrocytes, microglia, and damaged neurons within the injured CNS (Gyoneva and Ransohoff, 2015). Nevertheless, these results suggest that Müller glia, which we derived from donors with no known retinal diseases, have the potential to contribute to the expression of these factors during retinal gliosis.

Semiquantitative arrays showed that the proinflammatory enzyme myeloperoxidase was highly downregulated in the PVR retina as compared with the normal retina. This enzyme is involved in catalysing the formation of reactive oxygen species that contribute to inflammation. High levels have been associated with cardiovascular disease (Nicholls and Hazen, 2005) and multiple sclerosis (Gray et al., 2008), highlighting the contribution of this enzyme to retinal gliosis. Although this enzyme was observed in Müller cell lysates, the significance of its downregulation during retinal gliosis is yet to be defined. It may be possible that the decrease observed in the PVR retina was merely due to retinal cell death, or to regulatory mechanisms triggered during retinal inflammation that inhibit the production of this enzyme. Other factors found to be downregulated in the gliotic retina as compared with the normal retina include angiogenin, the matrixin enzyme MMP‐9, and proinflammatory C‐reactive protein. Angiogenin is a secreted ribonuclease that promotes RNA transcription and cell growth. It was first identified as an angiogenic factor produced by tumor cells and is thought to promote cell and tissue adaptation (Lai et al., 2015). That this protein is highly downregulated in the gliotic retina as compared with the normal retina, may reflect the limited proliferative and regenerative ability of the cells present in the gliotic tissue. MMP‐9 is involved in the degradation of the extracellular matrix (ECM) that promotes tissue remodelling (Vandooren et al., 2013), therefore the downregulation of this enzyme in PVR retina may contribute to the progression of gliosis by stabilising the ECM. C‐reactive protein (CRP) is an acute‐phase protein of hepatic origin that increases following interleukin‐6 secretion from macrophages and T‐cells (Worthmann et al., 2015). This protein is rapidly produced in response to inflammatory signals but quickly declines after a short period (Povoa, 2002), for which it can be suggested that its low levels in the gliotic retina may reflect the chronicity of PVR. Because C‐reactive protein was not shown to be present in Müller lysates, this may indicate that other retinal inflammatory cells may be producing this protein during gliosis.

The factor found to be the most abundant in the Müller cell lysates as judged by semiquantitative analysis, and has not been previously detected in Müller glia, was the plasminogen activator inhibitor 1 (serpin E1). Serpin E1, an inhibitor of fibrinolysis and matrix metalloproteinases, has been implicated in inflammatory diseases contributing to the progression of fibrosis (Loskutoff and Quigley, 2000). However, it was not found to be one of the predominant factors in the lysates of normal and gliotic human retina. Another matrix‐associated protein, the extracellular matrix metalloproteinase inducer (EMMPRIN), was also found to be abundant in Müller glia and although it was present at relatively high levels in the retinal lysates, there was no difference in expression between the gliotic and normal retina. That not all the factors examined were detected in both, isolated Müller glia and retinal specimens may be due to the fact that Müller cells in culture may de‐differentiate and lose many of their typical physiological and functional features upon *in vitro* culture. Although in gliotic PVR retina there is severe loss of retinal neurons and predominance of reactive Müller glia expressing GFAP and CRALBP (Charteris et al., 2007; Ghosh and Johansson, 2012; Wickham et al., 2007), it is possible that factors expressed by Müller glia may be under‐represented in the retinal samples due to the presence of other retinal cell types.

TGFβ signalling is well known for its role in promoting Müller glia proliferation (Close et al., 2005), and is thought to contribute to the gliotic response observed in retinal degenerations (Guerin et al., 2001). Quantitative analysis of the three TGFβ isoforms identified TGFβ1 as the predominant isoform produced by Müller glia *in vitro*, its values being on average 38% higher than those of TGFβ2. In contrast, TGFβ2 was the predominant isoform detected in normal retina, being 2.7 times the levels of TGFβ1. In addition, TGFβ2 was the only isoform to be significantly upregulated in the PVR retina as compared with the normal retina (*P* < 0.05). It has been documented that Müller glia in culture produce TGFβ2 and that this cytokine inhibits the proliferation of retinal endothelial cells (Yafai et al., 2014). It is of interest that our results showed that Müller glia produces comparable levels of TGFβ2 to those previously reported (Yafai et al., 2014). However, a comparison between the three different isoforms of TGFβ production by Müller glia has not been previously shown. From the present observations it is possible to suggest that some of the TGFβ2 produced by Müller glia may account for the high levels present in the gliotic retina, but it is also likely that cells other than Müller glia may constitute an additional source of this cytokine within the gliotic retina. This imbalance might contribute to the progression of the gliotic response and merits further investigations.

In conclusion, this study showed that the pattern of expression of the majority of cytokines and proinflammatory factors found to be significantly elevated in lysates of PVR retina as compared with normal human retina parallels the pattern of expression of these factors expressed by Müller glia in culture. That the majority of factors identified in cultured Müller glia by semi quantitative and quantitative analyses were detected in retinal specimens (87 and 96%, respectively), and that 70% of the quantitated factors were significantly upregulated in the gliotic retina, as compared with normal retina, strongly suggest that Müller glia is an important source of cytokines and growth factors associated with retinal gliosis in PVR. Targeting the production of these factors by Müller glia may constitute a valid approach to prevent neural damage during many retinal diseases and this merits further investigations.
